# Acceptance as a Foundational Skill for Managing Ambiguous Loss in Dementia Caregiving

**DOI:** 10.1093/geroni/igaf122.1641

**Published:** 2025-12-31

**Authors:** Gavin Green, Josey Batura, Heather Kelley, Elizabeth Fauth

**Affiliations:** Utah State University, Logan, Utah, United States; Utah State University, Logan, Utah, United States; Utah State University, Logan, Utah, United States; Utah State University, Logan, Utah, United States

## Abstract

**Background:**

Dementia caregiving presents unique challenges, particularly ambiguous loss, in which caregivers witness the gradual psychological absence of their loved ones despite their continued physical presence.

**Objectives:**

This paper has three primary aims: (1) to conceptualize acceptance as a cognitive foundation that shapes how caregivers perceive, interpret, and respond to stress; (2) to examine the role of acceptance in fostering emotional resilience, highlighting its influence on psychological flexibility, stress mitigation, and caregiving effectiveness; and (3) to introduce acceptance as a clinical heuristic, providing a structured framework for therapeutic interventions and psychoeducational programs.

**Theoretical Framework & Approach:**

This theoretical analysis integrates Family Stress Theory (FST), specifically the ABCX and Family Adjustment and Adaptation Response (FAAR) models, with a synthesis of empirical literature on acceptance-based interventions such as Acceptance and Commitment Therapy (ACT).

**Key Contributions:**

We propose that fostering acceptance enhances psychological flexibility, reduces caregiver stress and burnout, and improves caregiving effectiveness. By positioning acceptance as a cognitive and clinical foundation, this paper provides a structured framework for guiding evidence-based caregiver interventions.

**Clinical Implications:**

We underscore the importance of acceptance-based psychoeducational programs, structured interventions, and therapeutic approaches in caregiver support.

**Conclusion:**

As dementia prevalence rises, equipping caregivers with evidence-based tools for resilience is essential for sustaining well-being and long-term caregiving effectiveness.

